# Correction: Development of a novel complex inflammatory bowel disease mouse model: Reproducing human inflammatory bowel disease etiologies in mice

**DOI:** 10.1371/journal.pone.0324178

**Published:** 2025-05-12

**Authors:** The PLOS One Staff

## Notice of Republication

An incomplete, earlier version of this article was published in error. The publisher apologises for the error. This article was republished on April 22, 2025, to correct for this error. Please download the article again to view the correct version. The originally published, uncorrected article and the republished, corrected article are provided here for reference.
