# Supplementary material for: Development of a novel complex inflammatory bowel disease mouse model: Reproducing human inflammatory bowel disease etiologies in mice
Source: PLoS One. 2024 Nov 21;19(11):e0311310. doi: 10.1371/journal.pone.0311310 (PMC11581264; doi:10.1371/journal.pone.0311310)
Supplement: S1 Raw image — (PDF) [file pone.0311310.s001.pdf]

S1_raw-images

Development of a novel complex inflammatory bowel disease mouse model: reproducing human inflammatory bowel disease etiologies in mice

Sun-Min Seo[1], Na-Won Kim[1], Eun-Seon Yoo[1], Ji-Hun Lee[1], Ah-Reum Kang[1], Han-Bi Jeong[1], Won-Yong Shim[1], Dong-Hyun Kim[1], Young-Jun Park[1], Kieun Bae[2], Kyong-Ah Yoon[2], Yang-Kyu Choi[1*]

[1] Department of Laboratory Animal Medicine, College of Veterinary Medicine, Konkuk University, Seoul, Republic of Korea
[2] Department of Veterinary Biochemistry, College of Veterinary Medicine, Konkuk University, Seoul, Republic of Korea

***Corresponding author:** yangkyc@konkuk.ac.kr (YKC)

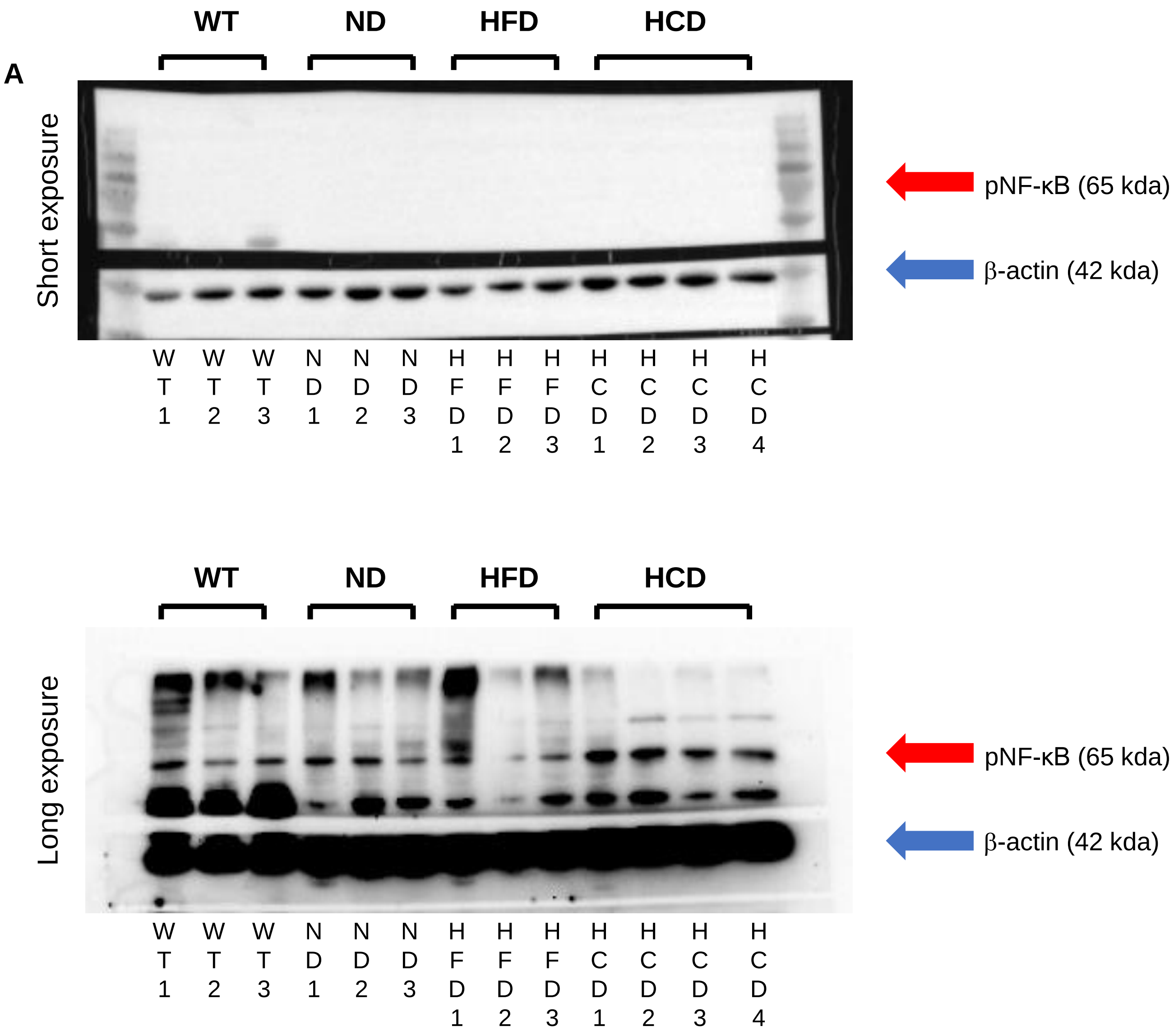

Uncropped images of blot presented in Fig 6A

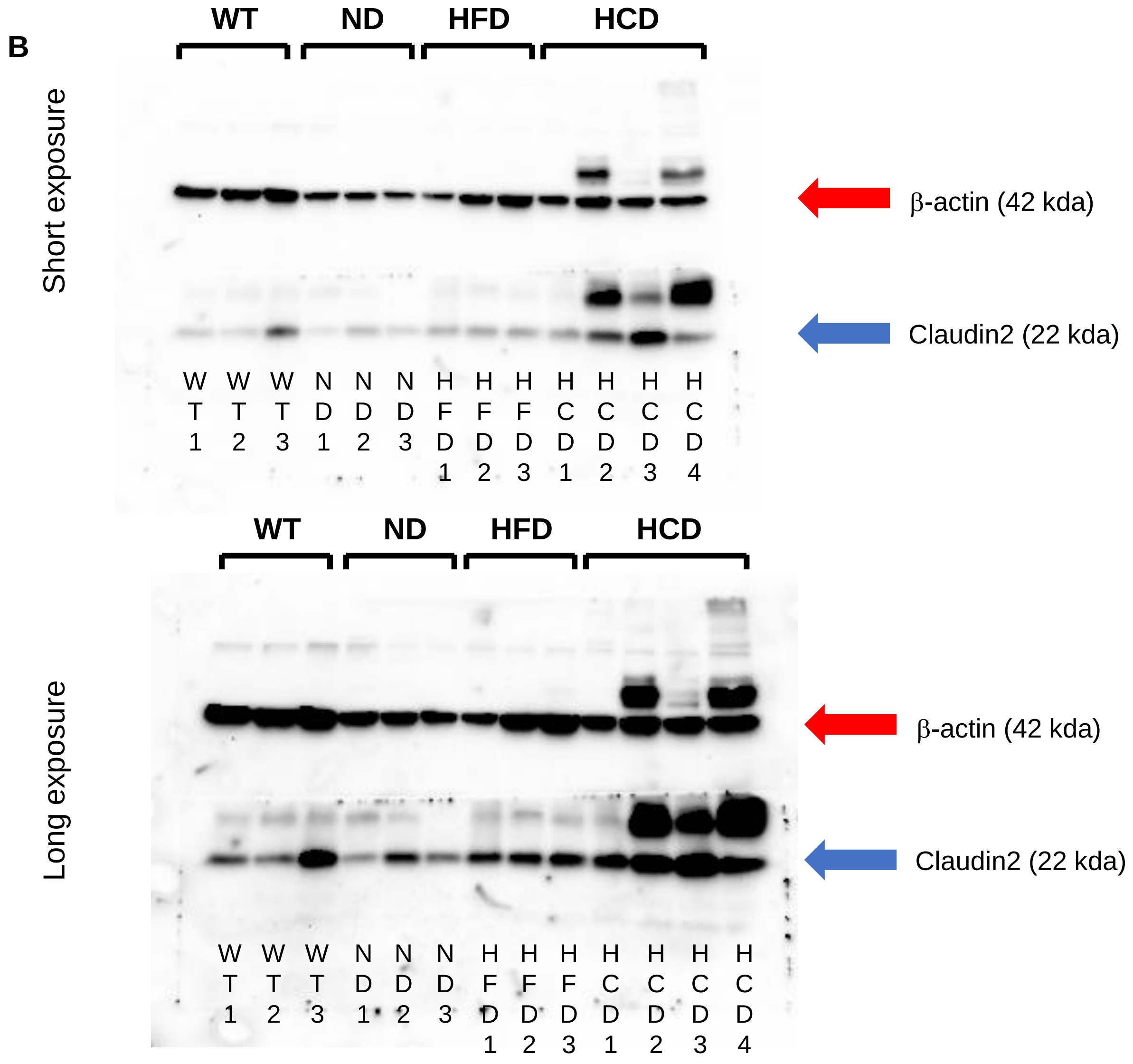

Uncropped images of blot presented in Fig 6B
