# Supplementary material for: Development of a novel complex inflammatory bowel disease mouse model: Reproducing human inflammatory bowel disease etiologies in mice
Source: PLoS One. 2024 Nov 21;19(11):e0311310. doi: 10.1371/journal.pone.0311310 (PMC11581264; doi:10.1371/journal.pone.0311310)
Supplement: Attachment — Submitted filename: PLOS ONE Review.pdf [file pone.0311310.s002.pdf]

PLOS ONE Review

Title: Development of novel complex inflammatory bowel disease mouse models: reproducing various human inflammatory bowel disease etiologies in mice.

In this study, the authors have developed the mouse model of IBD using various human IBD etiologies. However, many major concerns need to be addressed.

Major points:

- A change in the myeloperoxidase enzyme activity must support the change in the colon length.
- A complete picture of the Swiss roll must be provided along with the magnified inserts. The scale bar must be included in the picture.
- What does the bar diagram Fig 2B indicating the histopathological scoring indicate?
- The Fig2C bar diagram showing HFD and HCD standard deviation (SD) is very high. With this high SD, having a statistical significance in what is depicted is unrealistic. Therefore, statistics analysis must be revisited.
- To evaluate the reliability of the newly established IBD model, several parameters of key anti-inflammatory activity must be assessed. These include LI-6, IL-1B, TNF-alpha, and CXCL-10 protein concentration changes, preferably through Western Blot analysis.
- One of the key proinflammatory transcription factor that is activated is NF-κB; therefore, authors must investigate its expression of it.
- One of the key events that is affected is the breach of the tight junction. Therefore, colon permeability along with tight junction protein expression must be investigated before it can be acceptable as a model that mimics IBD with complex etiology.
