# Supplementary material for: Development of a novel complex inflammatory bowel disease mouse model: Reproducing human inflammatory bowel disease etiologies in mice
Source: PLoS One. 2024 Nov 21;19(11):e0311310. doi: 10.1371/journal.pone.0311310 (PMC11581264; doi:10.1371/journal.pone.0311310)
Supplement: Attachment — Submitted filename: Response to Reviewers.docx [file pone.0311310.s004.docx]

Pradeep Dudeja

Academic Editor

*PLOS ONE*

Dear Dr. Dudeja

Subject: Development of a novel complex inflammatory bowel disease mouse model: reproducing human inflammatory bowel disease etiologies in mice [PONE-D-24-05316R1]

Thank you for inviting us to submit a revised draft of our manuscript entitled, "Development of a novel complex inflammatory bowel disease mouse model: reproducing human inflammatory bowel disease etiologies in mice" to PLOS ONE. We appreciate the time and effort you and the reviewers have dedicated to providing insightful feedback to strengthen our paper. We are pleased to resubmit our article for further consideration, having incorporated the detailed suggestions provided. We believe that our edits and the responses below adequately address all the issues and concerns raised by you and the reviewers.

To facilitate your review of our revisions, the following is a point-by-point response to the questions and comments delivered in your letter dated 12 August 2024.

**Comments from Reviewer #1:**

The authors have done a good job in revising the manuscript and responding to this reviewer’s concerns. Thank you. There are a few questions

1. Page 14, Line 265-66: Can you comment on whether the HFD group was statistically different from WT or ND in mRNA or protein for IL-10?

A: Thank you for providing these insights. Although the WT and ND groups show lower Il-10 levels in both mRNA and protein analyses, no significant differences were observed compared to the HFD group. These results may be attributed to the use of nonparametric tests due to the limited sample size. We have included these additional results (p.13 line 257-260, p.14 line 279-282) and discussion (p.21 line 437-440) in our revised manuscript.

2. Page 16-17: Line 332-334: The two sentences appear contradictory – does Enterococcaceae increase in the WT group as suggested by the 2nd sentence?

A: We agree that our expression was ambiguous. We intended to explain that the relative abundance of Enterococcaceae increased in the ND, HFD, and HCD groups as factors known to influence IBD were applied, whereas the WT group deviated from this trend. The relative abundance of Enterococcaceae in the WT group was higher than in the ND and HFD groups. We have included this additional explanation in our revised manuscript (P.17-18 line 358-361). Additionally, we also revised the sentences concerning Ruminococcaceae and Lactobacillaceae to avoid potential confusion. (P.17 line 356-358)

3. Page 19 line 396-397: As written here it is unclear whether the change in IL-10 in HFD is associated with worsening or resolving the disease. In responses to the previous review (#8), the authors provide a clearer explanation and I recommend that language should be included here instead. “We concluded that IL-10 upregulation in the HFD group indicates the resolution of self-limiting inflammation, not becoming chronic form. This information is written in (p.19 line 395-400).”

A: Thank you for your suggestion. We agree that our expression was confusing, so we revised our manuscript to be more obvious (p. 20 line 423-425).

4. Page 32, Line 410: “This result indicates…..” is an incomplete sentence and should be deleted.

A: Thank you for your advice. As your advice, we deleted the incomplete sentence.

**Comments from Reviewer #3:**

The authors have addressed most of the concerns raised by the reviewers. However, the authors should consider the following suggestions:

1. Previous studies have shown an increase in the abundance of Proteobacteria (phylum associated with wide variety of pathogens) in CD patients (Ref#62). Figure 8A shows an increase in the abundance of Proteobacteria in HCD group compared to WT, ND and HFD groups. However, the authors failed to elaborate on this aspect in the revised manuscript. Moreover, further explanation on this aspect would be more convincing to establish that HCD model is good for the study of human CD.

A: Thank you for providing these insights. In addition to the increased abundance of Proteobacteria in the HCD group, we also discussed the reduced abundance of Actinobacteriota, which is known to be decreased in CD and UC patients. These results are presented in our revised result (p.17 line 335-341) and discussion (p.21 line 453-457) sections, along with the newly added Fig. 8B.

2. Any specific reason as to why HCD gp was not compared to WT and ND groups in Figs. 4D and 5D?

A: Thank you for your insightful feedback. Although the HCD group showed lower levels of Il-10 in both mRNA and protein analyses, no statistically significant differences were observed compared to the WT and ND groups, so we initially did not include this data. However, following the recommendations of Reviewer 1 and Reviewer 3, we have now added these results to the revised manuscript (p.13 line 257-260, p.14 line 279-282). As mentioned in our response to Reviewer 1's Question 1, the lack of statistical significance may be attributed to the use of non-parametric tests due to the limited sample size.

3. Please be consistent. Also compare HCD gp with ND gp in Figs. 4B, 4E, 4F, 6C and 6D.

A: As in our response to Question 2, no statistically significant differences were observed in Fig. 4B, 4E, 4F, 6C, and 6D, so we initially did not mention these results. However, following the reviewers' recommendations, we have now included a comparison of expression levels and significance between the HCD group and the ND, WT, and HFD groups in Fig. 4, Fig. 5, and Fig. 6 in the revised manuscript. (p. 13 line 243-255, p. 14 line 264-278, p. 15 line 301-306)

4. Did the authors examine NFκB (p65) protein levels in nuclear fraction of colon lysates? If yes, NFκB (p65) levels should be normalized to a nuclear protein, Lamin B1

A: We normalized p65 activation to β-actin because we aimed to assess the overall phosphorylation status of p65 by examining its protein levels in total lysates without distinguishing between the cytoplasm and nucleus. However, while preparing our response to the revision, we realized that our manuscript did not sufficiently mention the phosphorylation of NF-κB, and we had incorrectly described the antibody used. We have now corrected these details in our revised manuscript. (p. 9 line 148, p. 15 line 300, Fig. 6A, Fig 6C and supplemental figure)

Again, thank you for giving us the opportunity to strengthen our manuscript with your valuable comments and queries. We have worked hard to incorporate your feedback and hope that these revisions persuade you to accept our submission.

Sincerely yours,

Yang-Kyu Choi
